# Supplementary material for: A deep learning ECG model for identification and localization of occlusion myocardial infarction
Source: Nat Commun. 2026 May 13;17:4336. doi: 10.1038/s41467-026-73023-1 (PMC13171952; doi:10.1038/s41467-026-73023-1)

Supplementary Methods

PTB-XL external validation set

The PTB-XL is a publicly available database of 21,837 10-second 12-lead ECGs annotated with 71 different ECG statements.[13, 14] From PTB-XL, we included ECGs that had been evaluated by at least one PTB-XL cardiologist (validated_by_human=True) and with no electrode problems reported (empty electrodes_problem). This resulted in 16,065 ECGs, and from these we (before running any models) extracted:

- 63 STEMI ECGs. In total, 98 ECGs are annotated as likely acute myocardial infarctions (infarction_stadium1="Stadium I" or infarction_stadium2="Stadium I"). A senior cardiologist (JS) reviewed the ECGs for the presence of a ST-elevation and 63 ECGs with a definite ST elevation-pattern were kept.
- 279 LBBB ECGs. All ECGs labeled as complete LBBB (CLBBB:100) with available age and sex information were kept.
- 197 normal ECGs. In total, 200 likely normal controls without myocardial infarction were randomly drawn (empty infarction_stadium1 and empty infarction_stadium2 and NORM:100). Only a random subset of 200 was drawn to be able to complete a manual review within reasonable time. A senior cardiologist (JS) reviewed the ECGs for the presence of a ST-elevation and 197 ECGs without a definite ST elevation-pattern were kept.

Age and sex was extracted from the PTB-XL database. Age was missing for one STEMI ECG and imputed with the mean age of STEMIs in PTB-XL. Age was further normalized using the mean and standard deviation from the training dataset.

12SL diagnosis statements

From each GE MUSE ECG of the SwED database, we extracted all automatic ECG diagnosis statements from Marquette 12SL. An ECG with the following statements (in Swedish) were labeled as STEMI: "** ** AKUT MYOKARDINFARKT ** **" and "** ** AKUT MI/STEMI ** **". The model of the present study outputs predicted probabilities. When thresholding the probabilities to "call" a given outcome many aspects should be considered, including disease prevalence, risk vs benefit, health economics, healthcare load, and ethics. No such analysis has been performed. In this analysis we set a threshold that resulted in a comparable number of false positives to the 12SL statements.

Supplementary Tables

Supplementary Table 1. Number of ECGs per outcome class and set in the SwED study sample. The percent out of all ECGs in the given set/column is provided within parentheses. OMI, occlusion myocardial infarction; STEMI, ST-elevation myocardial infarction; NSTEMI, non-ST-elevation myocardial infarction; LM/LAD, left main coronary artery+left anterior descending artery; LCX, left circumflex artery; RCA, right coronary artery; Test (rand), random test set; Test (temp), temporal test set.

	**Training**	**Validation**	**Test (rand)**	**Test (temp)**
**Control**	410,434 (98.88)	48,699 (99.16)	48,680 (99.10)	26,697 (98.68)
Without perimyocarditis	409,303 (98.61)	48,590 (98.94)	48,566 (98.87)	26,606 (98.35)
With perimyocarditis	1,131 (0.27)	109 (0.22)	114 (0.23)	91 (0.34)
**nOMI**	3,416 (0.82)	296 (0.60)	339 (0.69)	228 (0.84)
NSTEMI	2,812 (0.68)	248 (0.50)	278 (0.57)	187 (0.69)
STEMI	604 (0.15)	48 (0.10)	61 (0.12)	41 (0.15)
**OMI**	1,236 (0.30)	115 (0.23)	104 (0.21)	128 (0.47)
NSTEMI, LM/LAD	93 (0.02)	20 (0.04)	7 (0.01)	18 (0.07)
NSTEMI, LCX	198 (0.05)	11 (0.02)	10 (0.02)	10 (0.04)
NSTEMI, RCA	157 (0.04)	10 (0.02)	11 (0.02)	7 (0.03)
STEMI, LM/LAD	346 (0.08)	37 (0.08)	34 (0.07)	40 (0.15)
STEMI, LCX	99 (0.02)	10 (0.02)	7 (0.01)	14 (0.05)
STEMI, RCA	343 (0.08)	27 (0.05)	35 (0.07)	39 (0.14)

Supplementary Table 2. Performance metrics of the model in the two test sets in SwED. Sample size is given by each test set and outcome label together with percent out of the given test set in Supplementary Table 1. Performance metrics are presented together with 95% confidence intervals from a non-parametric bootstrap. Arrows indicate direction of better performance. We compute the metrics as class *vs.* all other (OvA). For the Brier score and ECE, a multi-class calculation is provided, calculated for the ten MI sub-classes. MI, myocardial infarction; OMI, occlusion MI; STEMI, ST-elevation myocardial infarction; NSTEMI, non-ST-elevation myocardial infarction; LM/LAD, left main coronary artery+left anterior descending artery; LCX, left circumflex artery; RCA, right coronary artery; AP, Average Precision or equivalently Area Under the Precision-Recall curve; ECE, Expected Calibration Error; Test (rand), random test set; Test (temp), temporal test set.

Metric	Outcome	Test (rand)	Test (temp)
C-statistic (↑)	Control, without perimyocarditis	0.8762 (0.8593,0.8924)	0.8906 (0.8709,0.9086)
	Control, with perimyocarditis	0.9078 (0.8725,0.9387)	0.8696 (0.8195,0.9147)
	nOMI, NSTEMI	0.8542 (0.8290,0.8774)	0.8696 (0.8426,0.8964)
	OMI, NSTEMI, LM/LAD	0.9488 (0.8952,0.9900)	0.8940 (0.7597,0.9816)
	OMI, NSTEMI, LCX	0.8320 (0.6455,0.9549)	0.9782 (0.9522,0.9970)
	OMI, NSTEMI, RCA	0.9122 (0.8095,0.9978)	0.9945 (0.9903,0.9980)
	nOMI, STEMI	0.9467 (0.9085,0.9768)	0.9593 (0.9310,0.9823)
	OMI, STEMI, LM/LAD	0.9768 (0.9383,0.9984)	0.9926 (0.9871,0.9974)
	OMI, STEMI, LCX	0.9648 (0.8941,0.9999)	0.9949 (0.9904,0.9984)
	OMI, STEMI, RCA	0.9792 (0.9569,0.9948)	0.9866 (0.9706,0.9977)
	LBBB	0.9855 (0.9798,0.9904)	0.9903 (0.9793,0.9968)
	MI	0.8907 (0.8735,0.9078)	0.9206 (0.9027,0.9363)
	OMI	0.9534 (0.9255,0.9761)	0.9764 (0.9574,0.9903)
	OMI LM/LAD	0.9712 (0.9323,0.9945)	0.9617 (0.9181,0.9902)
	OMI LCX	0.8851 (0.7694,0.9661)	0.9856 (0.9716,0.9959)
	OMI RCA	0.9636 (0.9302,0.9880)	0.9877 (0.9729,0.9973)
	nOMI	0.8699 (0.8487,0.8904)	0.8846 (0.8606,0.9070)
	STEMI	0.9638 (0.9433,0.9814)	0.9819 (0.9726,0.9899)
	NSTEMI	0.8580 (0.8348,0.8810)	0.8827 (0.8570,0.9062)
AP (↑)	Control, without perimyocarditis	0.9978 (0.9974,0.9982)	0.9970 (0.9962,0.9977)
	Control, with perimyocarditis	0.2252 (0.1468,0.3070)	0.2329 (0.1400,0.3461)
	nOMI, NSTEMI	0.1068 (0.0756,0.1451)	0.1661 (0.1201,0.2276)
	OMI, NSTEMI, LM/LAD	0.0094 (0.0011,0.0603)	0.0411 (0.0131,0.1034)
	OMI, NSTEMI, LCX	0.0019 (0.0007,0.0053)	0.1668 (0.0308,0.5161)
	OMI, NSTEMI, RCA	0.0963 (0.0313,0.2429)	0.0323 (0.0141,0.0908)
	nOMI, STEMI	0.1703 (0.1055,0.2761)	0.1304 (0.0711,0.2082)
	OMI, STEMI, LM/LAD	0.5391 (0.3841,0.7026)	0.5650 (0.4171,0.7289)
	OMI, STEMI, LCX	0.1995 (0.0733,0.5834)	0.2756 (0.0832,0.5495)
	OMI, STEMI, RCA	0.5437 (0.3770,0.7080)	0.5919 (0.4339,0.7330)
	LBBB	0.8755 (0.8351,0.9116)	0.9229 (0.8935,0.9483)
	MI	0.3681 (0.3218,0.4145)	0.5383 (0.4804,0.5876)
	OMI	0.4975 (0.4027,0.5951)	0.5892 (0.5069,0.6770)
	OMI LM/LAD	0.5245 (0.3659,0.6731)	0.4827 (0.3502,0.6249)
	OMI LCX	0.0890 (0.0154,0.2837)	0.2757 (0.1295,0.4682)

	OMI RCA	0.5319 (0.3813,0.6710)	0.6012 (0.4633,0.7359)
	nOMI	0.1695 (0.1311,0.2141)	0.2057 (0.1576,0.2602)
	STEMI	0.5795 (0.4930,0.6597)	0.6861 (0.6115,0.7589)
	NSTEMI	0.1252 (0.0922,0.1673)	0.2206 (0.1667,0.2817)
Brier **(↓)**	Control, without perimyocarditis	0.0088 (0.0083,0.0093)	0.0109 (0.0102,0.0118)
	Control, with perimyocarditis	0.0021 (0.0018,0.0023)	0.0029 (0.0025,0.0033)
	nOMI, NSTEMI	0.0054 (0.0052,0.0056)	0.0063 (0.0060,0.0066)
	OMI, NSTEMI, LM/LAD	0.0001 (0.0001,0.0001)	0.0007 (0.0006,0.0007)
	OMI, NSTEMI, LCX	0.0002 (0.0002,0.0002)	0.0003 (0.0003,0.0004)
	OMI, NSTEMI, RCA	0.0002 (0.0002,0.0002)	0.0003 (0.0002,0.0003)
	nOMI, STEMI	0.0011 (0.0010,0.0012)	0.0014 (0.0013,0.0015)
	OMI, STEMI, LM/LAD	0.0004 (0.0003,0.0006)	0.0008 (0.0006,0.0011)
	OMI, STEMI, LCX	0.0001 (0.0001,0.0001)	0.0005 (0.0004,0.0005)
	OMI, STEMI, RCA	0.0004 (0.0003,0.0006)	0.0008 (0.0006,0.0010)
	LBBB	0.0090 (0.0068,0.0118)	0.0029 (0.0024,0.0035)
	MI	0.0069 (0.0065,0.0074)	0.0083 (0.0077,0.0091)
	OMI	0.0014 (0.0012,0.0016)	0.0028 (0.0024,0.0031)
	OMI LM/LAD	0.0005 (0.0004,0.0007)	0.0014 (0.0011,0.0017)
	OMI LCX	0.0003 (0.0003,0.0004)	0.0008 (0.0007,0.0008)
	OMI RCA	0.0006 (0.0005,0.0007)	0.0010 (0.0007,0.0012)
	nOMI	0.0063 (0.0060,0.0066)	0.0074 (0.0070,0.0078)
	STEMI	0.0016 (0.0014,0.0019)	0.0025 (0.0021,0.0029)
	NSTEMI	0.0059 (0.0056,0.0061)	0.0072 (0.0068,0.0075)
	Multi	0.0189 (0.0181,0.0197)	0.0249 (0.0238,0.0261)
ECE **(↓)**	Multi	0.0024 (0.0020,0.0032)	0.0055 (0.0049,0.0064)

Supplementary Table 3. Metrics of discriminative performance in the external validation sets CODE-II (Brazilian) and PTB-XL (European). Only MI labels for the presence of STEMI (yes/no) are available in these external validation sets and can be evaluated in addition to LBBB. In CODE-II, outcome-vs-rest comparisons are performed where the rest group consists of mostly normal ECGs. In PTB-XL, the outcome is compared with normal ECGs without any artifacts. Arrows indicate the direction of better performance. The C-statistic and average precision (AP) are provided.

Metric	Outcome	CODE-II	PTB-XL
C-statistic (↑)	STEMI	0.9872	0.9977
	LBBB	0.9870	0.9979
AP (↑)	STEMI	0.5406	0.9936
	LBBB	0.8733	0.9988

Supplementary Table 4. Over-/underrepresented diagnoses when comparing correct classifications with misclassifications for a given observed class, restricted to ECGs with a high predicted probability (Pr>0.5). Tested in an asymptotic general independence test with a two-sided alternative hypothesis. All diagnoses at time of the visit are included (all diagnosis positions). Three-character ICD10 codes were tested unless only the ICD10 block was available in the data. All results with a false discovery rate (Benjamini-Hochberg) < 0.01 are reported.

Truth	Mis-classification	Over/under represented diagnosis	OR
Control	nOMI	Intentional self-harm by hanging, strangulation and suffocation (X70)	335.0
Control	nOMI	Asphyxiation (T71)	297.2
Control	nOMI	Postprocedural disorders of genitourinary system, not elsewhere classified (N99)	245.0
Control	nOMI	Diseases of pulp and periapical tissues (K04)	191.4
Control	nOMI	Peptic ulcer, site unspecified (K27)	178.4
Control	OMI	Complications & ill-defined descriptions of heart disease (I51)	121.2
Control	nOMI	Postprocedural respiratory disorders, not elsewhere classified (J95)	111.9
Control	OMI	Cardiac arrest (I46)	81.6
Control	OMI	Acute myocarditis (I40)	79.9
Control	nOMI	Acute and subacute endocarditis (I33)	74.6
Control	nOMI	No diagnosis information (U99)	61.0
Control	nOMI	Vascular disorders of intestine (K55)	51.7
Control	nOMI	Other arthritis (M13)	48.9
Control	nOMI	Pregnant state, incidental (Z33)	48.9
Control	nOMI	Cardiac arrest (I46)	42.5
Control	OMI	Bipolar affective disorder (F31)	34.3
Control	nOMI	Streptococcal sepsis (A40)	32.3
Control	nOMI	Subarachnoid haemorrhage (I60)	32.3
Control	nOMI	Acute myocarditis (I40)	28.6
Control	nOMI	Other cerebrovascular diseases (I67)	28.0
Control	nOMI	Sequelae with surgical & medical care as external cause (Y88)	26.9
Control	OMI	Cardiomyopathy (I42)	26.2
Control	nOMI	Sarcoidosis (D86)	25.3
Control	OMI	Gastro-oesophageal reflux disease (K21)	24.1
Control	OMI	Symptoms and signs involving speech and voice (R47-R49)	24.1
Control	nOMI	Specific personality disorders (F60)	21.6
Control	OMI	Paroxysmal tachycardia (I47)	19.0
Control	nOMI	Acute renal failure (N17)	17.8
Control	nOMI	Other sepsis (A41)	13.3
Control	nOMI	Chronic ischaemic heart disease (I25)	10.6
Control	nOMI	Bacterial pneumonia, not elsewhere classified (J15)	8.3
Control	nOMI	Chronic kidney disease (N18)	7.7
Control	nOMI	Angina pectoris (I20)	6.2
Control	nOMI	Heart failure (I50)	5.3
Control	nOMI	Type 2 diabetes mellitus (E11)	5.0

Supplementary Table 5. Manual review of 20 misclassified ECGs. The upper section presents 10 false positives (Scenario 1), and the lower section shows 10 false negatives (Scenario 2). Diagnoses were reassigned by a consultant cardiologist.

**Scenario 1 - False Positives: Model predicted STEMI (probability > 0.9), originally labeled as Not STEMI**					
Predicted probability					Consultant Cardiologist Review
nOMI, STEMI	OMI, STEMI, LM/LAD	OMI, STEMI, LCX	OMI, STEMI, RCA	STEMI	
0.24	0.01	0.02	0.70	0.97	Left atrial enlargement, STEMI
0.42	0.54	0.00	0.01	0.97	STEMI
0.07	0.00	0.02	0.90	0.99	STEMI
0.34	0.54	0.01	0.01	0.90	ST elevation: pericarditis pattern
0.20	0.80	0.00	0.00	1.00	STEMI
0.20	0.78	0.00	0.00	0.98	Q wave pathological, STEMI
0.20	0.00	0.07	0.66	0.93	STEMI
0.28	0.63	0.00	0.01	0.92	STEMI
0.25	0.66	0.00	0.01	0.92	First degree AVB, left atrial enlargement, pathological Q wave, ST elevation (dyskinetic area), nonspecific intraventricular conduction delay
0.16	0.01	0.04	0.75	0.96	RBBB, STEMI
**Scenario 2 - False Negatives: Model predicted Not STEMI (probability < 0.1), originally labeled as STEMI**					
Predicted probability					Consultant Cardiologist Review
nOMI, STEMI	OMI, STEMI, LM/LAD	OMI, STEMI, LCX	OMI, STEMI, RCA	STEMI	
0.01	0.00	0.00	0.00	0.01	Left atrial enlargement, left anterior hemiblock, left ventricular hypertrophy,STEMI, PVC
0.01	0.01	0.00	0.00	0.02	Technical problem
0.00	0.00	0.00	0.00	0.01	ST elevation: pericarditis pattern
0.00	0.00	0.00	0.00	0.00	Sinus tachycardia, STEMI
0.00	0.00	0.00	0.00	0.00	Left anterior hemiblock, left atrial enlargement, left ventricular hypertrophy, technical problem
0.02	0.01	0.00	0.01	0.05	AF, STEMI
0.00	0.00	0.00	0.00	0.00	Left atrial enlargement, LVH, PVC
0.02	0.02	0.00	0.00	0.05	STEMI, Subendocardial ischemia
0.00	0.00	0.00	0.00	0.00	LBBB, STEMI
0.01	0.01	0.00	0.00	0.01	Technical problem

Supplementary Table 6. Clinical characteristics of the study sample. SwED study sample used in training, validation and test, stratified by control/nOMI/OMI outcome. Data are median (interquartile range) or percent out of the total number of ECGs of the column. *Prevalent disease based on any diagnosis position, inpatient and outpatient specialist care combined. **Combining troponin and high-sensitive troponin laboratory measurements from regional laboratory databases and the SWEDEHEART database. Maximum of all available measurements within the time window reported. Done separately for troponin I and T. ***Primary diagnosis from inpatient specialist care. OMI, occlusive myocardial infarction; nOMI, MI without OMI; ED, emergency department; NTproBNP, N-terminal pro-B-type natriuretic peptide.

	**Control**	**nOMI**	**OMI**
Number of ECGs	534,510	4,279	1,583
**Clinical characteristics at ED visit**			
Age	65 (48,78)	68 (59,76)	64 (56,73)
Male	47.6	69.9	79.9
Year	2012.0 (2009.0,2014.0)	2013.0 (2011.0,2015.0)	2013.0 (2011.0,2015.0)
**Common presenting complaints at ED visit**			
Chest pain	21.6	77.8	78.1
Difficulty breathing	14.3	9.1	4.6
Dizziness	7.0	0.4	0.9
Heart problem/arrhythmia	9.4	2.7	2.2
Circulatory arrest	0.1	1.2	4.6
Abdominal pain	4.7	1.7	2.3
**Cardiovascular diagnoses prior to ED visit***			
Myocardial infarction	8.8	19.1	14.0
Unstable angina	4.6	9.6	5.0
Ischemic heart disease	21.1	34.1	20.8
Stroke	9.1	6.7	5.6
Peripheral artery disease	7.0	10.4	5.2
Heart failure	16.2	11.8	8.7
Atrial fibrillation	19.8	10.9	7.3
Cardiovascular disease	58.8	61.7	49.3
**Drugs with >=1 dispensation within one year prior to ED visit**			
Renin-angiotensin system inhibitors	32.1	48.9	37.5
Calcium channel blockers	17.5	26.3	20.7
Beta-receptor blockers	35.8	43.7	32.3
Mineralocorticoid receptor antagonists	6.3	4.3	2.8
Diuretics	27.5	24.1	16.9
Anti-arrhythmic drugs	1.7	0.4	0.6
Statins	23.3	39.5	26.3
Anticoagulants	12.3	7.8	5.2
Antiplatelets	27.9	40.8	26.9
**Cardiac biomarkers within ED visit or coronary care unit hospitalization****			
Troponin I measured	1.0	9.3	11.1
Max troponin I (ng/L)	29.7 (29.7,40.0)	2350.0 (467.5,9425.0)	24200.0 (7200.0,54000.0)
Troponin T measured	35.7	88.9	91.0
Max troponin T (ng/L)	9.9 (5.0,19.0)	263.0 (81.0,863.0)	2100.0 (470.0,4880.0)

NT-pro-BNP measured	8.2	20.0	18.6
Max NT-pro-BNP (ng/L)	1350.0 (288.0,4360.0)	1510.0 (442.8,5100.0)	2460.0 (476.0,5142.5)
**Main diagnoses at admission*** **			
Myocardial infarction	0.0	98.4	98.4
Unstable angina	0.3	5.4	1.4
Ischemic heart disease	1.6	98.8	98.4
Stroke	2.1	0.4	0.8
Peripheral artery disease	0.4	0.4	0.1
Heart failure	3.5	1.5	1.5
Atrial fibrillation	5.9	0.8	0.2
Cardiovascular disease	19.0	99.6	99.7
**Mortality after ED visit**			
30-day all-cause death	3.4	3.7	7.1
30-day in-hospital all-cause death	2.5	3.2	6.1

Supplementary Table 7. Number of ECGs per outcome class in the InCor external validation set (São Paulo, Brazil), stratified by ECG vendor and overall.

Outcome	GE MUSE	Mortara	Total
Control	92	144	236
OMI	25	140	165
OMI-STEMI	1	28	29
OMI-NSTEMI	24	112	136

Supplementary Table 8. Metrics of discriminatory performance for OMI in the InCor external validation set (São Paulo, Brazil). OMI is compared vs control, stratified by ECG vendor (GE MUSE and Mortara) as well as overall. In InCor, 82% of the OMIs were NSTEMI, compared with 27% NSTEMI in SwED test set. Arrows indicate the direction of better performance. The C-statistic and average precision (AP) are provided.

Metric	Outcome	GE MUSE	Mortara	Total
**C-statistic (↑)**	OMI	0.8504	0.8277	0.8291
**AP (↑)**	OMI	0.6132	0.8431	0.7942

Supplementary Table 9. Metrics of discriminative performance for OMI-STEMI and OMI-NSTEMI in the InCor external validation set (São Paulo, Brazil). OMI-STEMI and OMI-NSTEMI are compared vs control, stratified by ECG vendor (GE MUSE and Mortara) as well as overall. Only one OMI-STEMI was available for GE MUSE, so performance was not assessed for that outcome and vendor. Arrows indicate the direction of better performance. The C-statistic and average precision (AP) are provided.

Metric	Outcome	GE MUSE	Mortara	Total
**C-statistic (↑)**	OMI-STEMI	NA	0.9593	0.9591
	OMI-NSTEMI	0.8288	0.8265	0.8201
**AP (↑)**	OMI-STEMI	NA	0.8874	0.8573
	OMI-NSTEMI	0.5244	0.8020	0.7123

Supplementary Table 10. STEMI vs prediction of STEMI, from 12SL (automatic diagnosis statements in the GE MUSE format) and the model of the present study. From 12SL no predicted probabilities are available, only called outcome labels. For the present study's model a probability cutoff of STEMI is set so that the proportion of false positives is comparable to 12SL. Results of truth vs prediction are presented for both SwED test sets combined. *6 ECGs out of 71 misclassified ECGs from the study model are correctly classified by 12SL; a manual review of those ECGs by a senior cardiologist (JS) revealed cases with unusual patterns, high heart rates, disturbances, and possible label noise (e.g. early repolarisation patterns), that could all be possible explanations for the confusion.

	**Truth**	**Prediction**	**Number**
**12SL**	No STEMI	No STEMI	75503
	STEMI	STEMI	133
	No STEMI	STEMI	402
	STEMI	No STEMI	138
**Study model**	No STEMI	No STEMI	75511
	STEMI	STEMI	200
	No STEMI	STEMI	394
	STEMI	No STEMI	71*

Supplementary Table 11. Rows represent STEMI vs prediction of STEMI, from 12SL among all with STEMI-OMI. *Pr(STEMI) is the median (IQR) predicted probability of STEMI from the study's model. **%STEMI is the proportion of ECGs predicted as STEMI by the study's model.

**Truth**	**Prediction (12SL)**	**N**	**Pr(STEMI), model***	**%STEMI, model****
STEMI-OMI	STEMI	98	0.96 (0.76,0.99)	95%
STEMI-OMI	No STEMI	71	0.16 (0.01,0.67)	59%

Supplementary Table 12. Stratified metrics of discriminative performance in the external validation set CODE-II (Brazilian). The outcome groups STEMI and LBBB are tested versus the rest group consisting of mostly normal ECGs. Strata include sex, age group (years), and comorbidity burden (defined as the presence of zero or at least one of seven pre-specified clinical conditions). Arrows indicate the direction of better performance. The C-statistic (C) and average precision (AP) are provided.

		Sex		Age group (years)						Comorbidities	
	**Outcome**	**Female**	**Male**	**18-39**	**40-49**	**50-59**	**60-69**	**70-79**	**≥80**	**0**	**≥1**
**C (↑)**	STEMI	0.9899	0.9827	0.9721	0.9880	0.9943	0.9887	0.9773	0.9792	0.9896	0.9851
**C (↑)**	LBBB	0.9943	0.9743	0.9368	0.9717	0.9829	0.9866	0.9869	0.9824	0.9837	0.9866
**AP (↑)**	STEMI	0.5656	0.5297	0.3062	0.5244	0.5582	0.5828	0.5616	0.5319	0.5379	0.5423
**AP (↑)**	LBBB	0.9205	0.8066	0.5414	0.7910	0.8587	0.8860	0.8952	0.8870	0.8482	0.8801

Supplementary Table 13. Sample size of strata in CODE-II, presented in Supplementary Table 12.

Strata	Stratum	STEMI	Not STEMI	LBBB
**Sex**	Female	925	1,229,103	22,341
	Male	1,810	852,038	18,039
**Age group (years)**	18-39	127	493,008	823
	40-49	275	372,876	2,018
	50-59	598	432,931	5,641
	60-69	836	402,109	10,663
	70-79	573	258,655	12,147
	≥80	326	121,562	9,088
**Comorbidities**	0	836	933,966	8,611
	≥1	1,899	1,147,175	31,769

Supplementary Figures

Supplementary Figure 1. Hierarchical organization of outcome classes used in this study. All MI and control sub-classes are mutually exclusive. LBBB is a co-diagnostic class that may co-occur with any other sub-class from the MI or control categories. Abbreviations: MI, myocardial infarction; OMI, occlusion MI; nOMI, non-occlusion MI; STEMI, ST-elevation MI; NSTEMI, non-ST-elevation MI; LM/LAD, left main coronary artery and left anterior descending artery; LCX, left circumflex artery; RCA, right coronary artery; LBBB, left bundle-branch block.

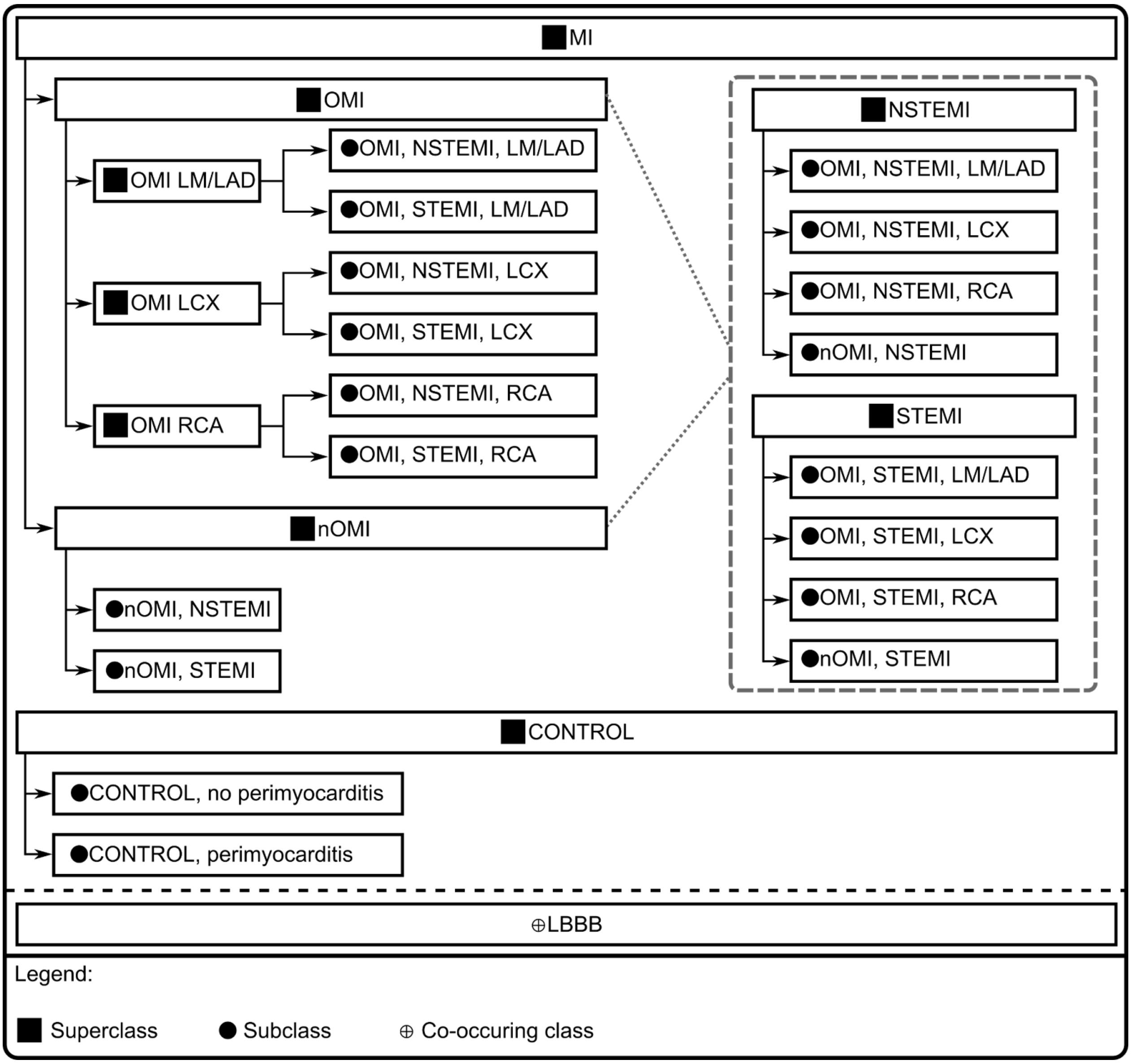

Supplementary Figure 2. Predicted probabilities of left bundle branch block (LBBB) from a machine learning model trained on Brazilian ECGs[15]. Separate panels for those with or without a prevalent diagnosis of LBBB in the SwED data. The dashed vertical line marks the lower cutoff (>=0.5) for classifying an ECG as LBBB. Note that all patients with prevalent LBBB will not have a diagnosis in the data (LBBB unknown/missed or diagnosis code not set). X-axis is on a log10 scale.

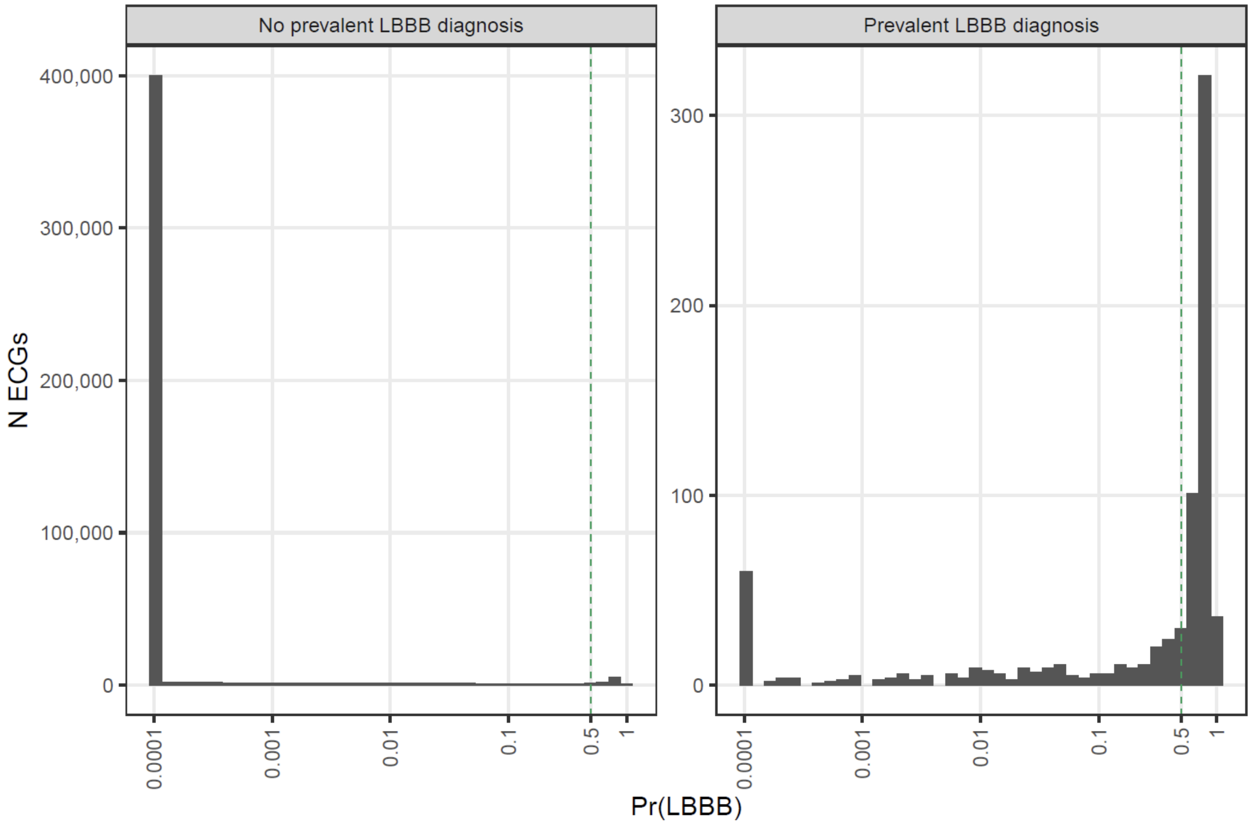

Supplementary Figure 3. Evaluation metrics were calculated in the validation set, for separate model fits with the BCE:CE weight ratio hyperparameter ranging from 0.05 to 20. Panel A: The C-statistic (reflecting model discrimination) was calculated in one-vs-rest for each of the 11 classes. Panel B: The Brier score (reflecting both the model discrimination and calibration) was calculated for LBBB (1 class) and the multi-class MI outcome (10 classes)

Panel A

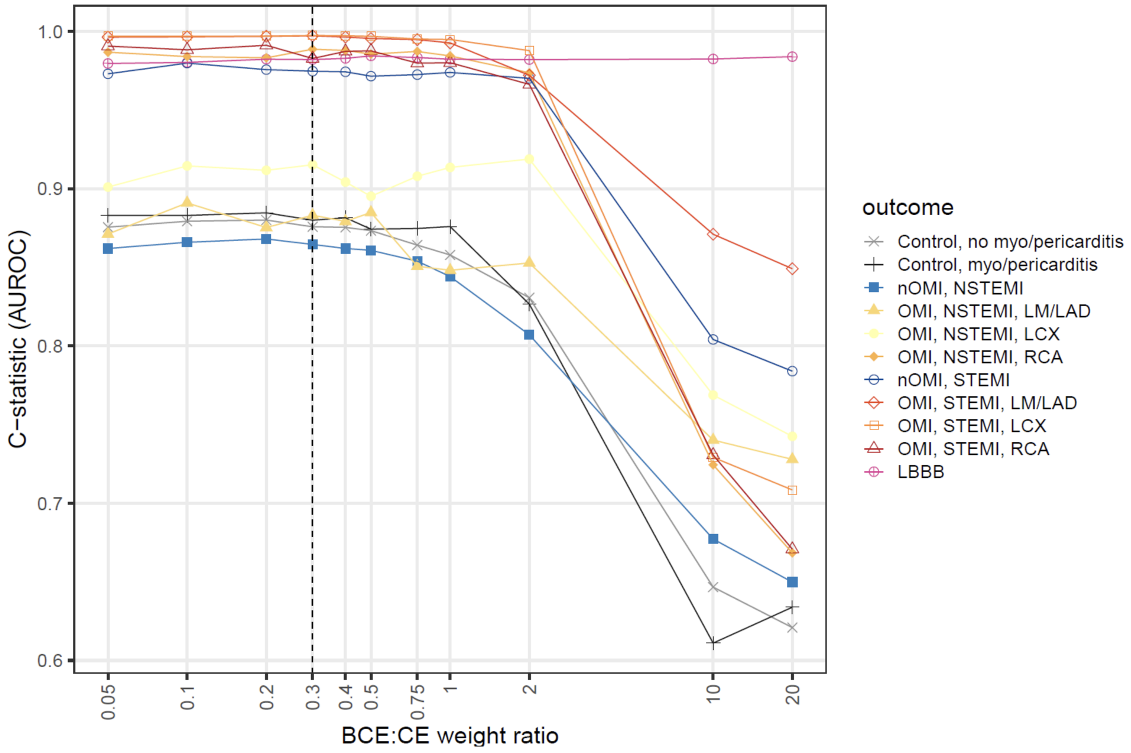

Panel B

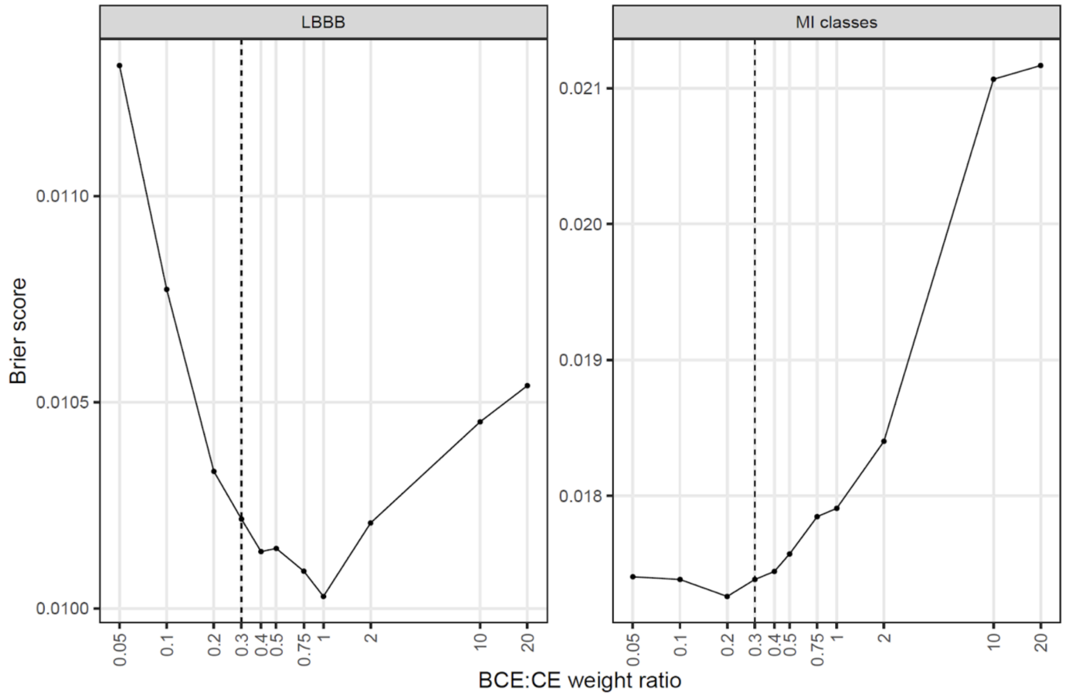

Supplementary Figure 4. Weighted total loss over epochs, across all five ensemble members.

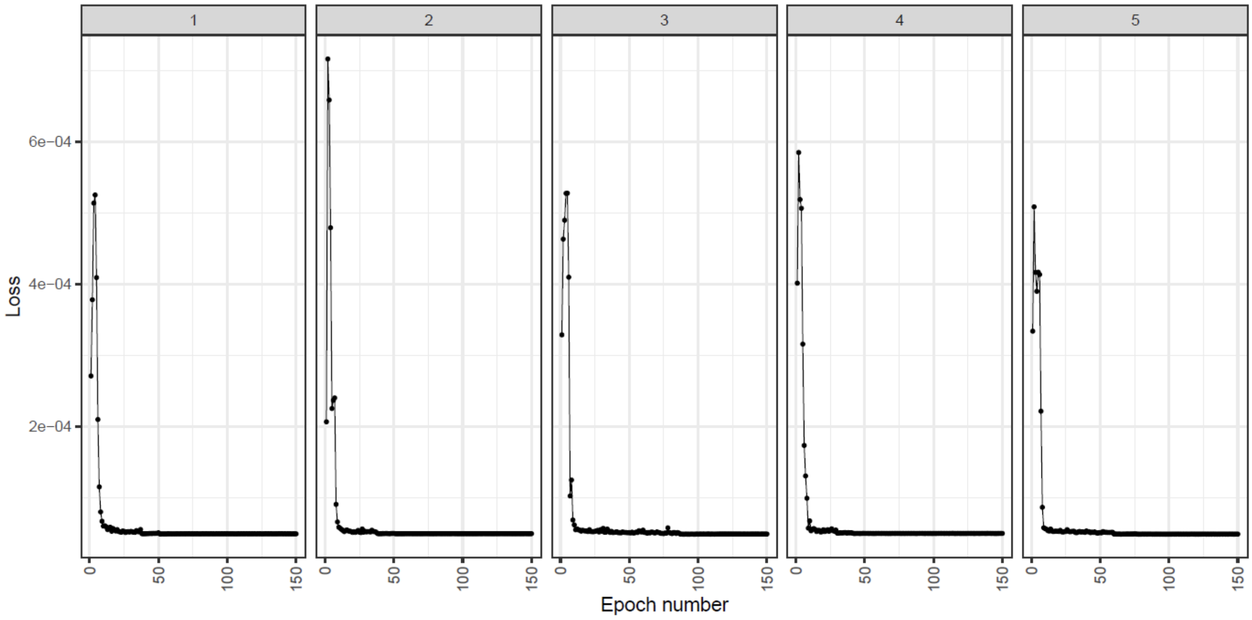

Supplementary Figure 5. Number of ECGs per year with separate panels for myocardial infarction (MI) with an urgent occlusion (OMI), MI without OMI (nOMI), and controls free from MI. MI cases from 2005-2006 were excluded due to missing database annotations needed for subcategorization as highlighted by the dotted vertical line. The dashed line represents the cutoff for the temporal test set.

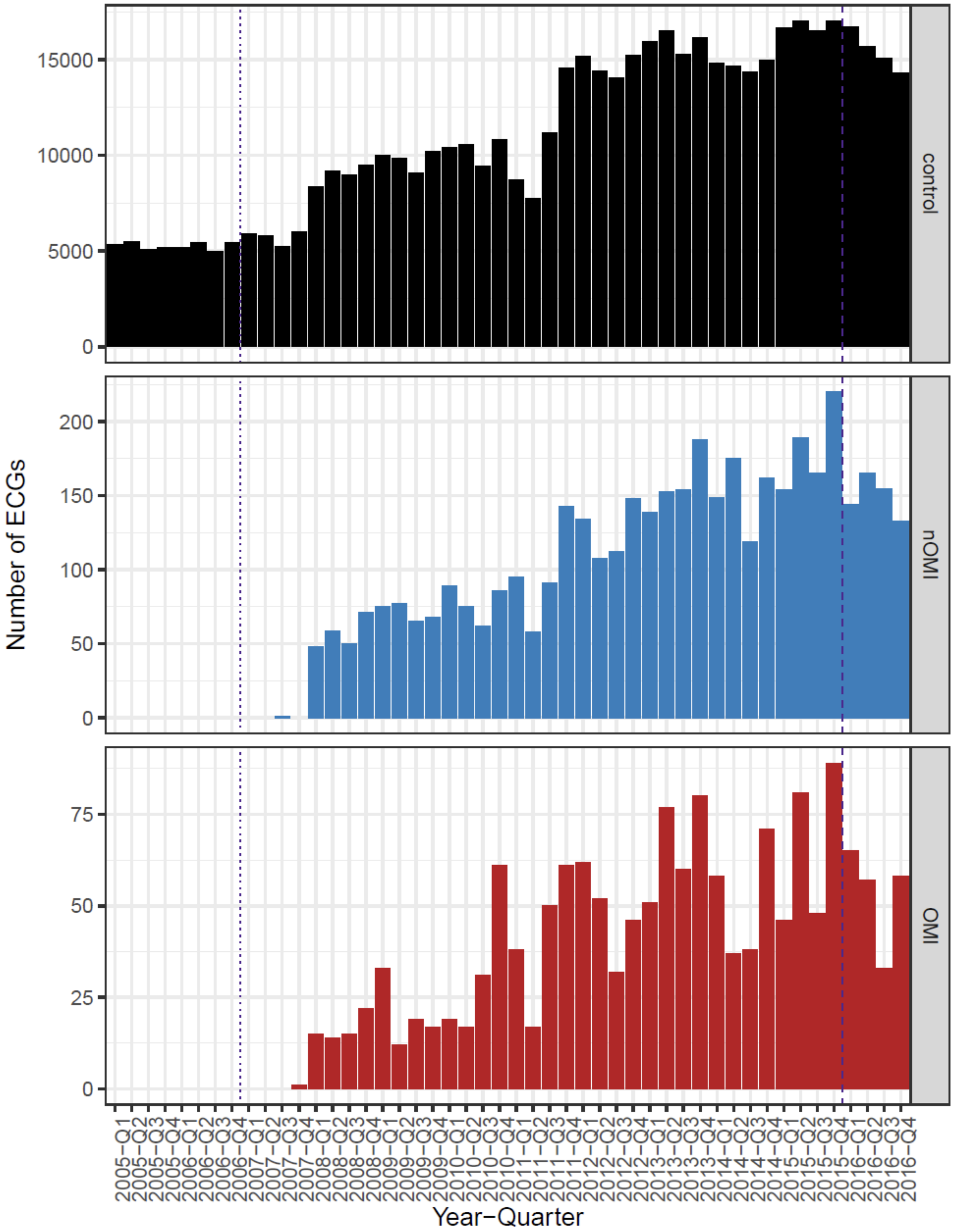

Supplementary Figure 6. Number of ECGs per year by outcome label, with separate panels for controls, MI, and LBBB. LBBB can co-occur with other classes. MI cases from 2005-2006 were excluded due to missing database annotations needed for subcategorization as highlighted by the dotted vertical line. The dashed line represents the cutoff for the temporal test set.

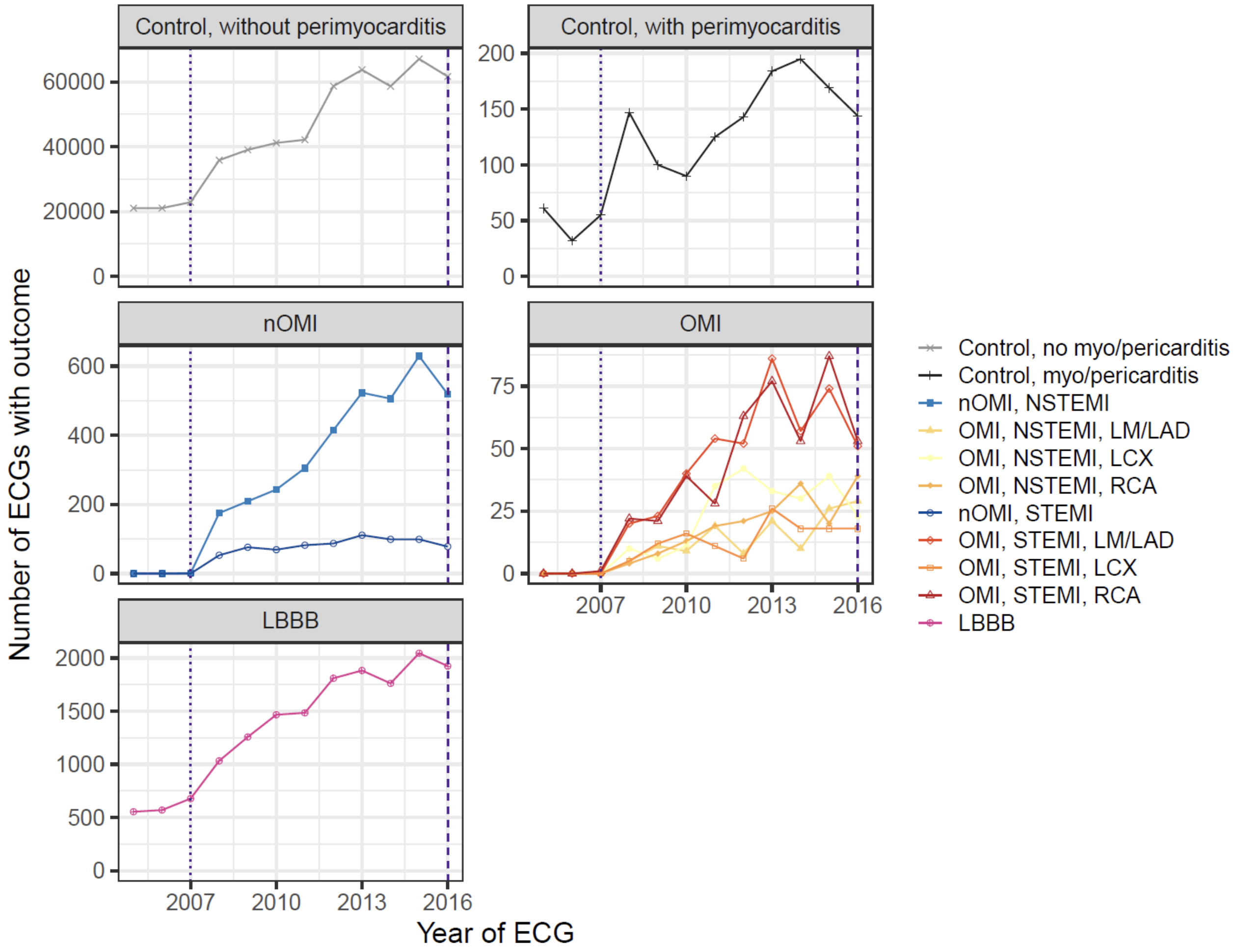

Supplementary Figure 7. Kaplan-Meier analysis of time to percutaneous coronary intervention (PCI) following the patients for up to 30 days after ED admission. Stratified by ED year tertiles and OMI. 95% confidence bands calculated from 2000 bootstrap draws.

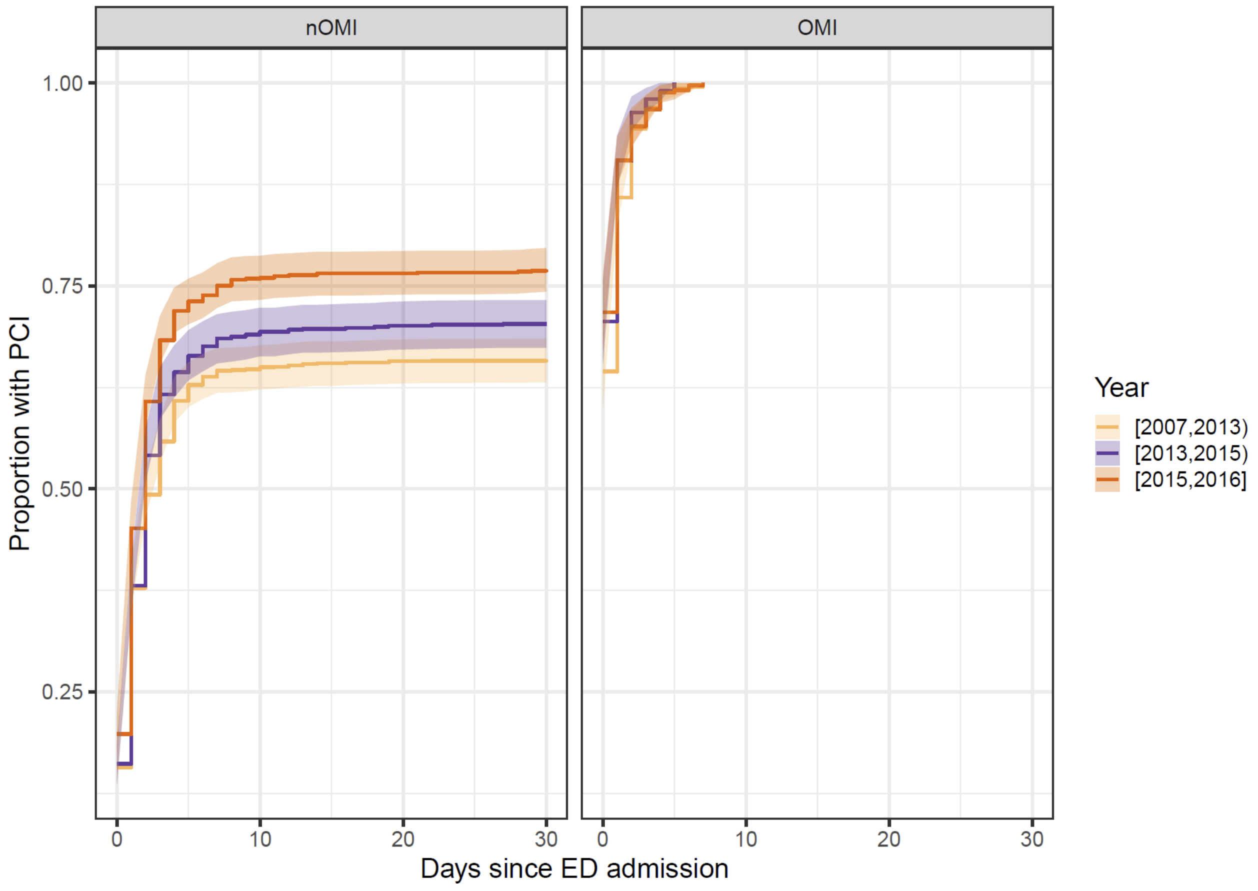

Supplementary Figure 8. Discriminative performance (C-statistic) when comparing a given class (x-axis) with all other classes in the random or temporal test set or combined. All predicted sub-classes of the model are included as well as the super-classes. 95% confidence intervals calculated from 2000 bootstrap draws. The dashed horizontal line represents a random guess.

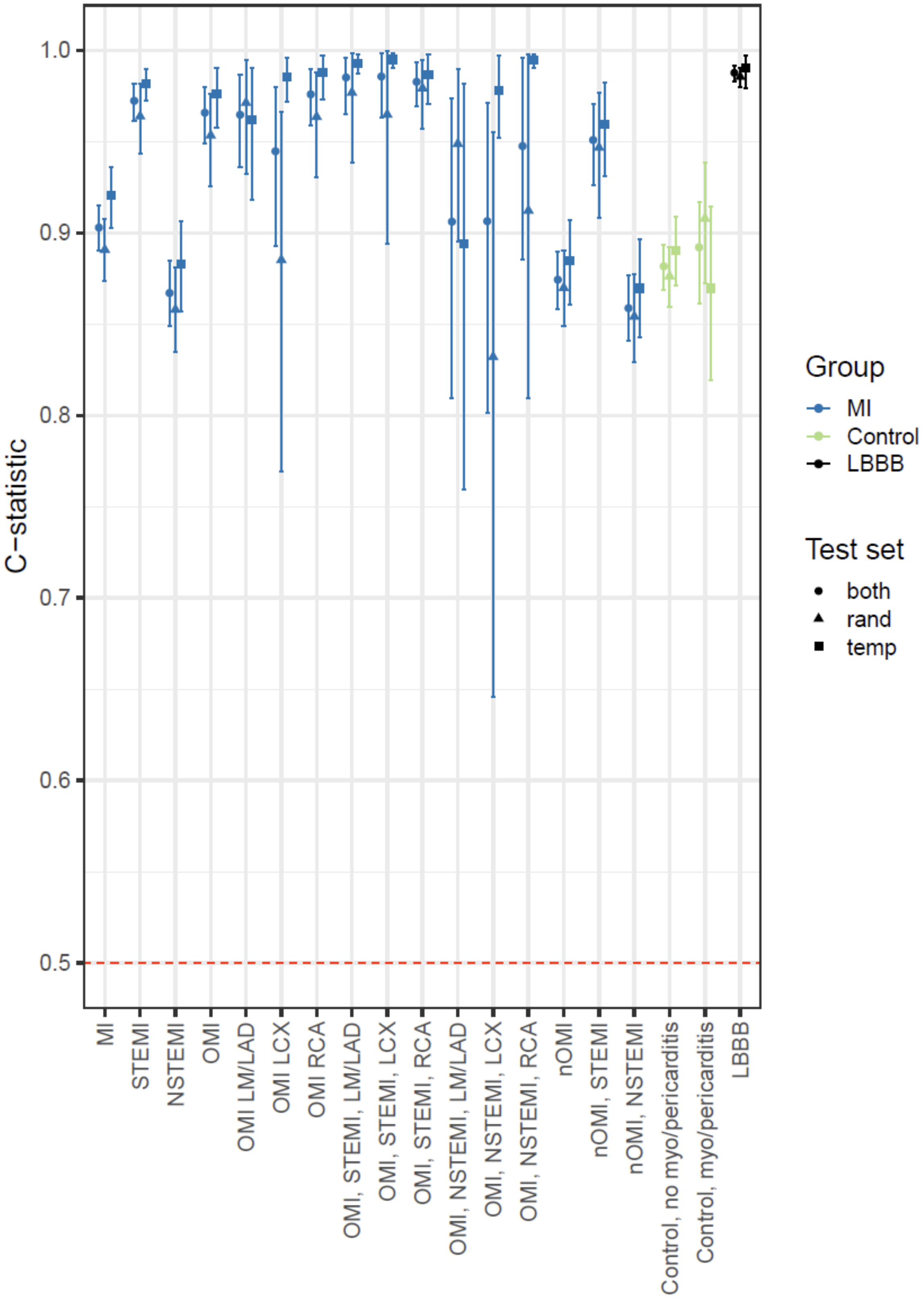

Supplementary Figure 9. Panel A showing receiver operating characteristics (ROC) curves, separately for each test set. Panel B showing precision-recall (PR) curves of each outcome (one versus rest), calculated from the combined test sets, using 50 fixed, evenly spaced bins, using linear interpolation between the points to avoid extremely jagged curves due to few cases in each outcome class.

Panel A)

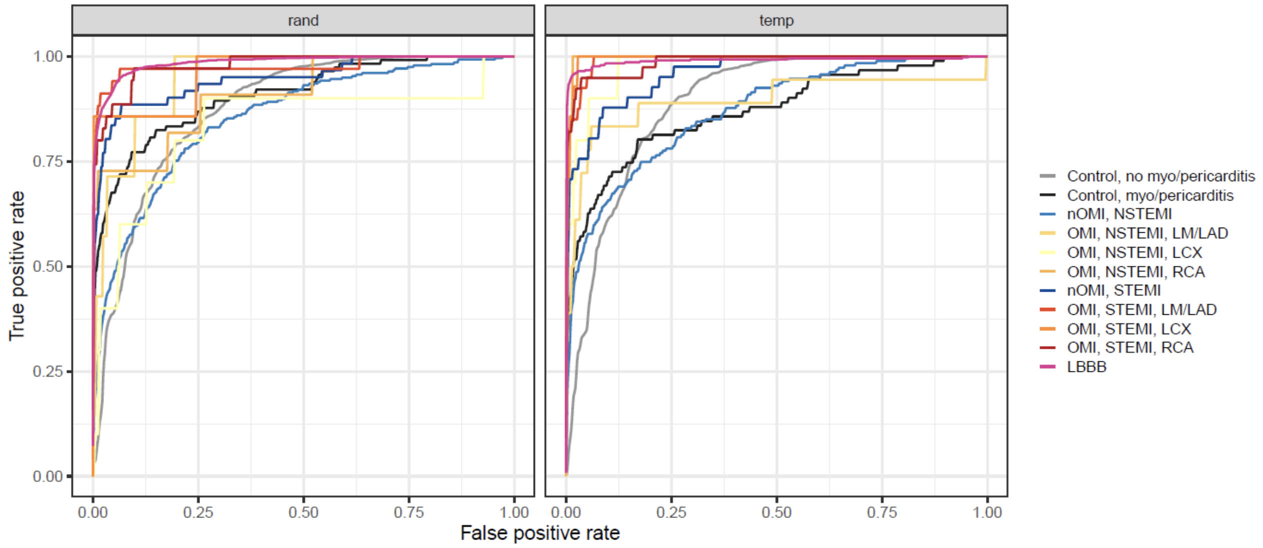

Panel B)

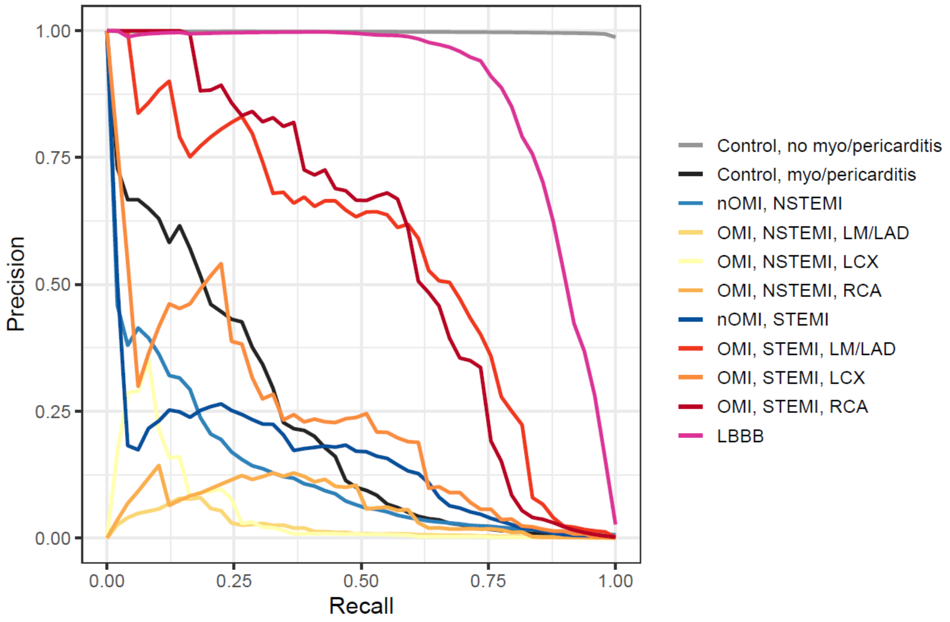

Supplementary Figure 10. Discriminative performance (C-statistic) in one versus one (OvO) comparisons. The predicted probability and truth label of outcome 2 (y-axis) is used in the calculation.

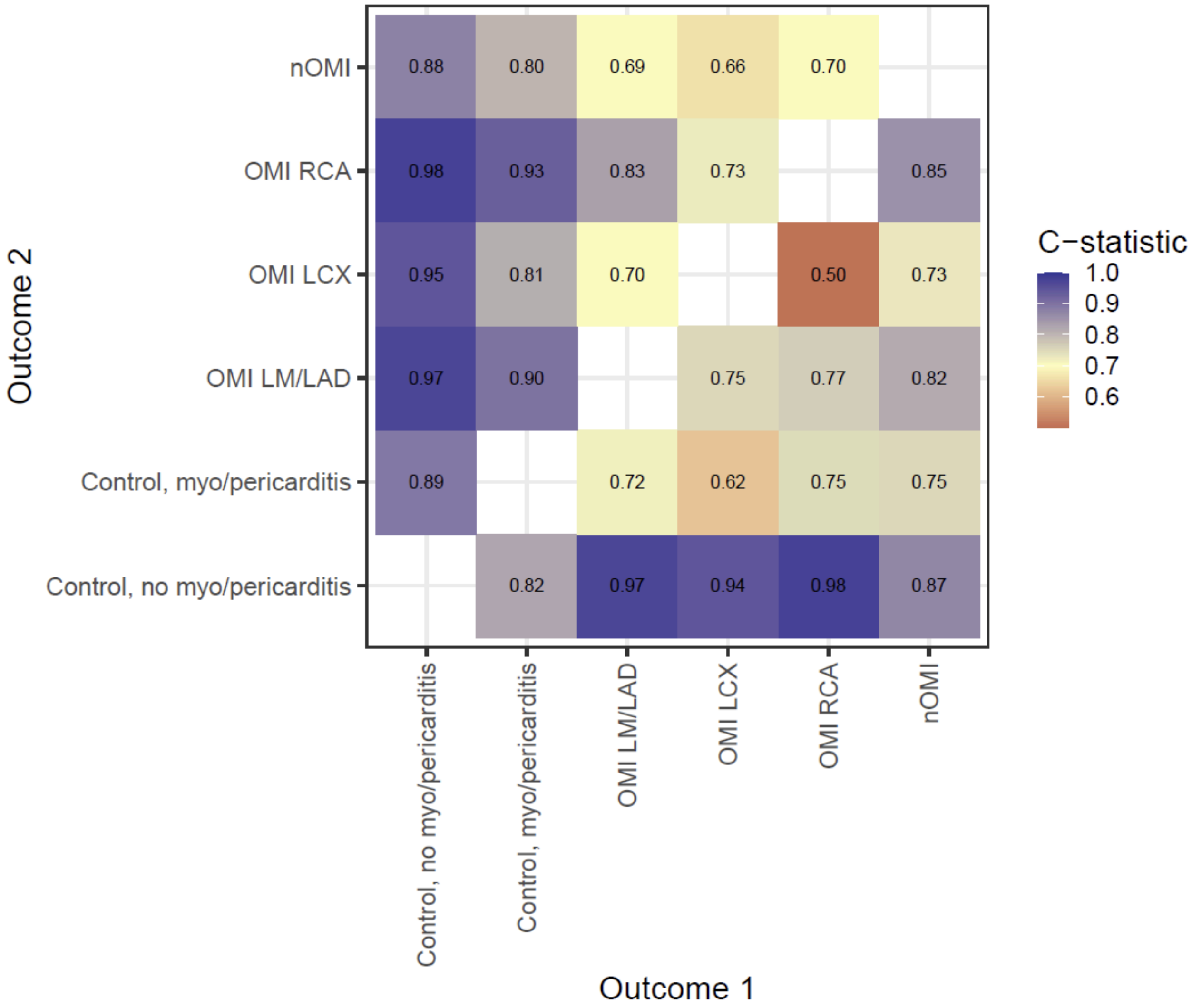

Supplementary Figure 11. Continuous calibration plots in both SwED test sets combined. Logistic calibration intercept and slope and a flexible smooth calibration curve estimated via a penalized thin-plate regression spline within a generalized additive model is presented. 95% confidence bands calculated from 2000 bootstrap draws.

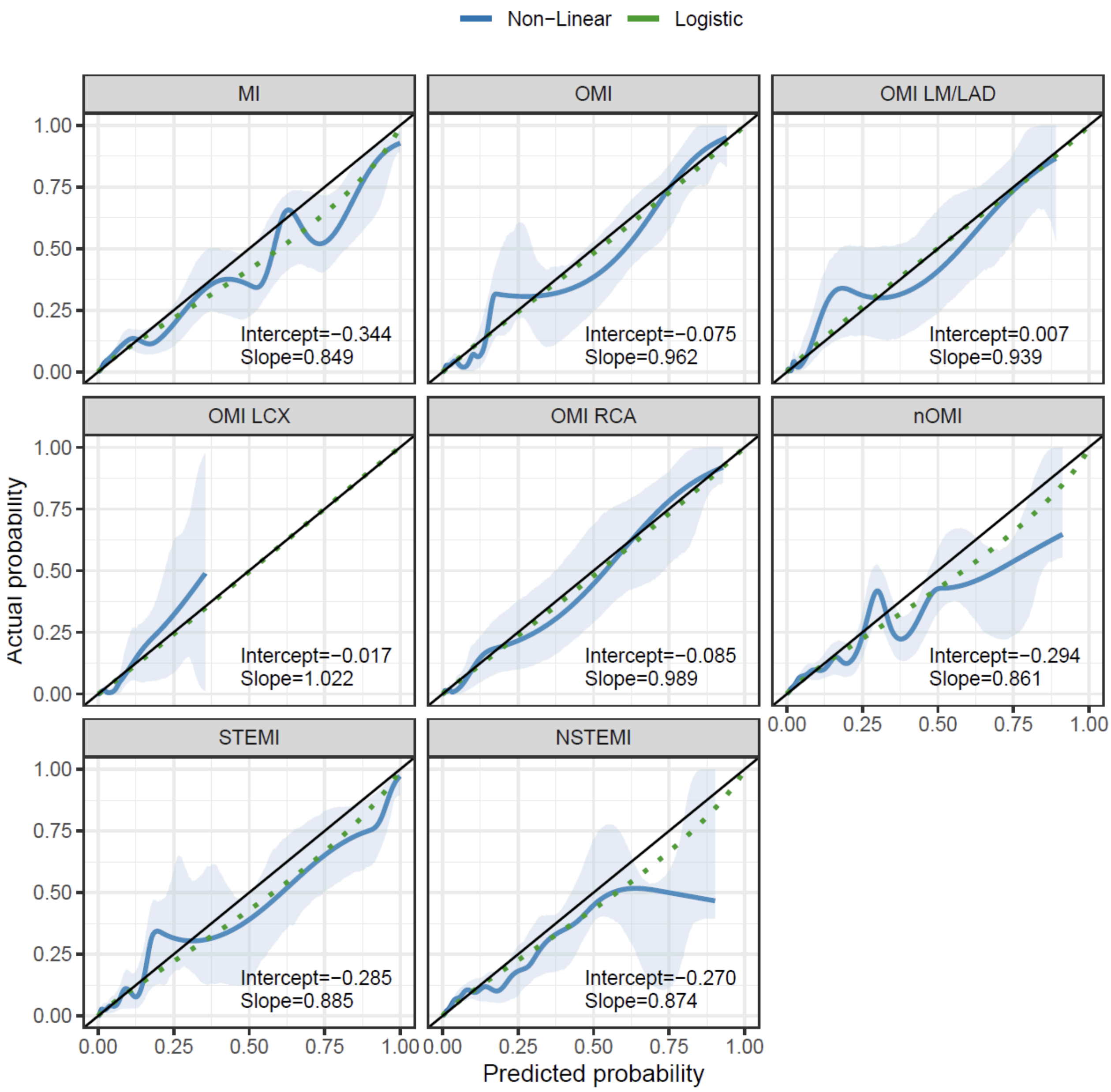

Supplementary Figure 12. Discriminative performance (C-statistic) when comparing a given class (color) with all other classes in the both SwED test sets combined, stratified by demographics, comorbidities, and technical factors, including age tertiles, sex, did the patient visit the emergency department at Karolinska Hospital (main source of data) or another emergency department in the Stockholm region, ECGs recorded using the most common machine type (MAC55) or not, ECGs recorded using the most common software (v237) or not, patient with a prevalent hospitalisation due to cardiovascular disease prior to the ED visit, patients with prevalent LBBB. 95% confidence bands calculated from 2000 bootstrap draws.

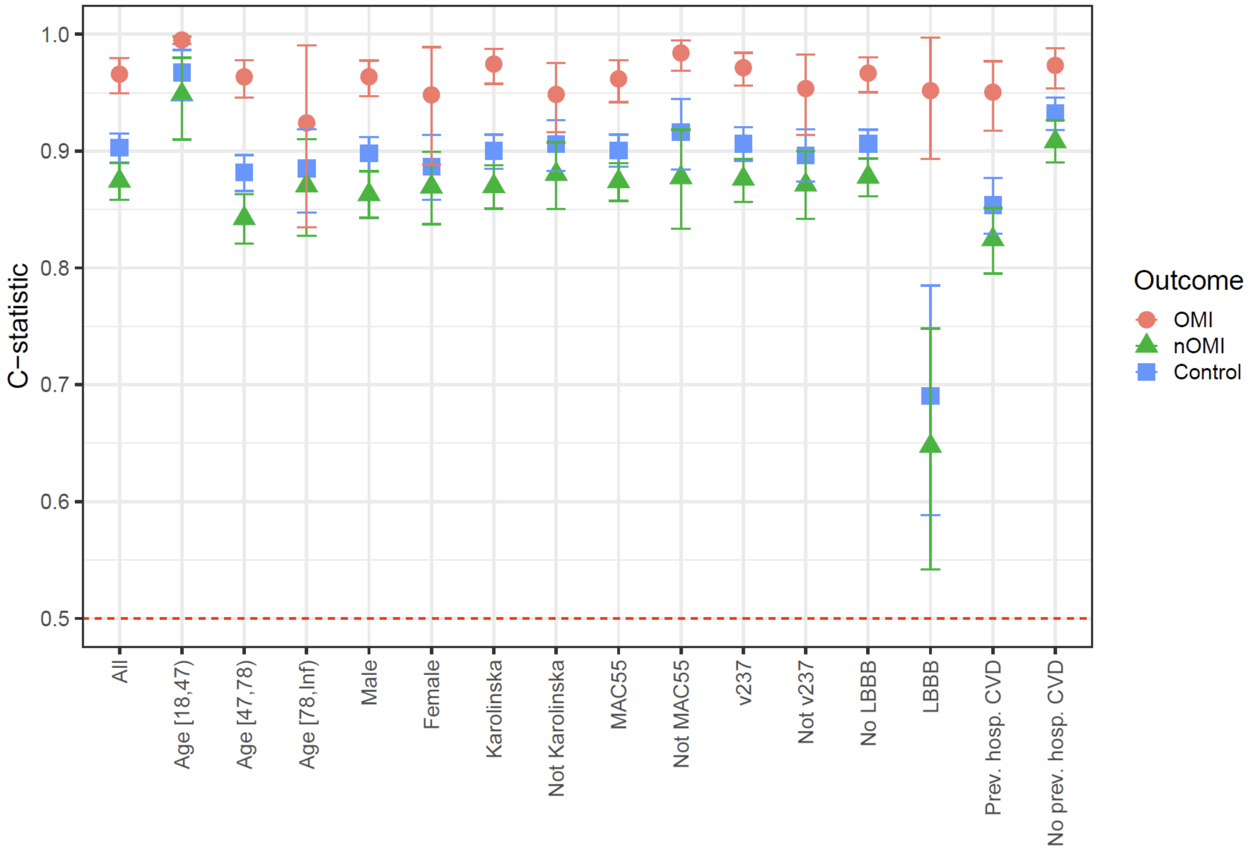

Supplementary Figure 13. Discriminative performance (C-statistic) of in both SwED test sets combined, stratified by year bins. 95% confidence intervals calculated from 2000 bootstrap draws.

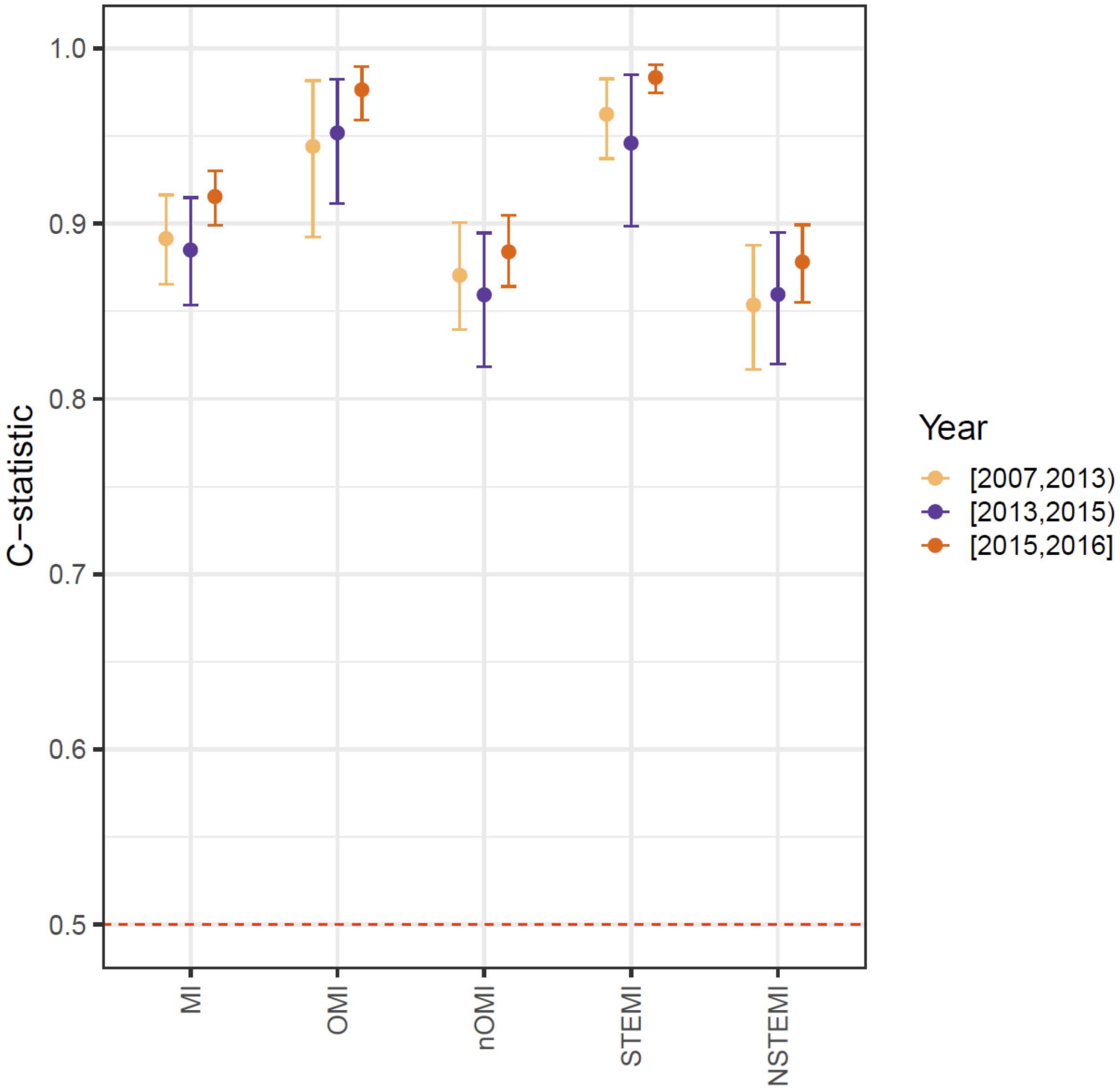

Supplement: Supplementary file 1 — Supplementary Information [file 41467_2026_73023_MOESM1_ESM.pdf]
