# Supplementary material for: A deep learning ECG model for identification and localization of occlusion myocardial infarction
Source: Nat Commun. 2026 May 13;17:4336. doi: 10.1038/s41467-026-73023-1 (PMC13171952; doi:10.1038/s41467-026-73023-1)
Supplement: Supplementary file 2 — Reporting summary [file 41467_2026_73023_MOESM2_ESM.pdf]

nature portfolio

Corresponding author(s): Johan Sundström

Last updated by author(s): Apr 1, 2026

Reporting Summary

Nature Portfolio wishes to improve the reproducibility of the work that we publish. This form provides structure for consistency and transparency in reporting. For further information on Nature Portfolio policies, see our Editorial Policies and the Editorial Policy Checklist.

Statistics

For all statistical analyses, confirm that the following items are present in the figure legend, table legend, main text, or Methods section.

n/a	Confirmed	
☐	☒	The exact sample size (*n*) for each experimental group/condition, given as a discrete number and unit of measurement
☐	☒	A statement on whether measurements were taken from distinct samples or whether the same sample was measured repeatedly
☐	☒	The statistical test(s) used AND whether they are one- or two-sided<br>*Only common tests should be described solely by name; describe more complex techniques in the Methods section.*
☐	☒	A description of all covariates tested
☐	☒	A description of any assumptions or corrections, such as tests of normality and adjustment for multiple comparisons
☐	☒	A full description of the statistical parameters including central tendency (e.g. means) or other basic estimates (e.g. regression coefficient) AND variation (e.g. standard deviation) or associated estimates of uncertainty (e.g. confidence intervals)
☐	☒	For null hypothesis testing, the test statistic (e.g. *F*, *t*, *r*) with confidence intervals, effect sizes, degrees of freedom and *P* value noted<br>*Give P values as exact values whenever suitable.*
☒	☐	For Bayesian analysis, information on the choice of priors and Markov chain Monte Carlo settings
☒	☐	For hierarchical and complex designs, identification of the appropriate level for tests and full reporting of outcomes
☒	☐	Estimates of effect sizes (e.g. Cohen's *d*, Pearson's *r*), indicating how they were calculated

Our web collection on statistics for biologists contains articles on many of the points above.

Software and code

Policy information about availability of computer code

Data collection	https://github.com/stefan-gustafsson-work/omi/<br>is now public
Data analysis	https://github.com/stefan-gustafsson-work/omi/<br>is now public

For manuscripts utilizing custom algorithms or software that are central to the research but not yet described in published literature, software must be made available to editors and reviewers. We strongly encourage code deposition in a community repository (e.g. GitHub). See the Nature Portfolio guidelines for submitting code & software for further information.

Data

Policy information about availability of data

All manuscripts must include a data availability statement. This statement should provide the following information, where applicable:

- Accession codes, unique identifiers, or web links for publicly available datasets
- A description of any restrictions on data availability
- For clinical datasets or third party data, please ensure that the statement adheres to our policy

The data that support the findings of this study are available from the Swedish Board of Health and Welfare and the included healthcare regions, but restrictions

apply to the availability of these data, which were used under license for the current study and cannot be publicly shared. Contact the corresponding author for any questions related to how to request the data.

Research involving human participants, their data, or biological material

Policy information about studies with human participants or human data. See also policy information about sex, gender (identity/presentation), and sexual orientation and race, ethnicity and racism.

Reporting on sex and gender	Sex as determined by the Swedish personal identifier number was used. Sex-stratified evaluation of model performance is included.
Reporting on race, ethnicity, or other socially relevant groupings	No variable that reliably captures race/ethnicity is available in the data. We do evaluate the model performance in various subgroups based on age, sex, pre-existing disease, hospital, year of visit etc. as described in the paper.
Population characteristics	All-comer emergency department (ED) patients >= 18 years old with a Swedish personal identifier number. EDs from the Stockholm region, Sweden with at least one ECG recording at time of the ED visit, between 2005 and 2016. Median age 65 years and 52% males in total. 534,510 controls, 4,279 non-occlusive myocardial infarction (MI) and 1,583 occlusive MI ECG recordings included.
Recruitment	All-comer ED patients from the given EDs and years, restricted to<br>1) >= 18 years old<br>2) have a Swedish personal identifier number<br>3) have an available ECG recording<br>4) MI cases that performed a angiography<br>Since Sweden is a country with publicly funded health care and since the inclusion criteria were kept as wide as possible, bias due to the recruitment criteria should be minimal. The good performance in other populations, e.g. Brazil, strengthens the argument that model performs well across multiple populations.
Ethics oversight	The study was approved by the Swedish Ethics Authority, accession numbers 2022-07108-01, 2023-05042-02, 2023-09-22, and 2024-04058-02 (SwED), and also approved by the Research Ethics Committee of the Universidade Federal de Minas Gerais, CAAE 85892325.1.0000.5149 (CODE-II) as well as the Research Ethics Committee of the Clinics Hospital, Heart Institute, University of São Paulo Medical School, CAAE 45070821.3.0000.0068 (InCor). Informed consent was waived by the Swedish Ethical Review Authority (on the basis of public interest, in accordance with GDPR Article 9(2)(j) and the Swedish Ethical Review Act [2003:460]), the Research Ethics Committee of the Universidade Federal de Minas Gerais, and the Research Ethics Committee of the Clinics Hospital, Heart Institute, University of São Paulo Medical School.

Note that full information on the approval of the study protocol must also be provided in the manuscript.

Field-specific reporting

Please select the one below that is the best fit for your research. If you are not sure, read the appropriate sections before making your selection.

☒ Life sciences ☐ Behavioural & social sciences ☐ Ecological, evolutionary & environmental sciences

For a reference copy of the document with all sections, see nature.com/documents/nr-reporting-summary-flat.pdf

Life sciences study design

All studies must disclose on these points even when the disclosure is negative.

Sample size	All ED patients with available ECG data:<br>540,372 ECGs from 465,471 ED visits and 225,824 patients<br>534,510 controls, 4,279 non-occlusive myocardial infarction (MI) and 1,583 occlusive MI ECG recordings included<br>Sample size for various subgroups are presented in detail in the paper.<br>No sample size calculation was performed a priori. This is all the data that is available from the included hospitals. Previous models with similar architecture have been able to train models with impressive performance with similar or lower sample size.
Data exclusions	In order to define all outcome categories the MI cases were required to have an angiography exam performed. This should restrict the data to a population that is highly relevant and for which the prediction model could in the future be used. Other exclusions were performed before we received the data, i.e. restriction to adults (>=18) with a Swedish personal identifier number. Myocardial infarction typically occurs in older individuals.
Replication	Internal validation of the prediction model using and random and temporal test split. External validation in three diverse replication sets (PTB-XL [European], CODE-II [Brazilian], and InCor [Brazilian]). Good prediction performance could be replicated across all these sets.
Randomization	The main study sample was split into training, validation, and test by a random draw at patient level. No patients overlap between the sets to prevent information leakage. Besides that, randomization is not applicable to the training of our model.
Blinding	This is retrospective data and the diagnoses have already been set when the data was collected. These are the outcomes we try to predict. Blinding is not applicable to this study.

Reporting for specific materials, systems and methods

We require information from authors about some types of materials, experimental systems and methods used in many studies. Here, indicate whether each material, system or method listed is relevant to your study. If you are not sure if a list item applies to your research, read the appropriate section before selecting a response.

Materials & experimental systems

n/a	Involved in the study
☒	☐ Antibodies
☒	☐ Eukaryotic cell lines
☒	☐ Palaeontology and archaeology
☒	☐ Animals and other organisms
☒	☐ Clinical data
☒	☐ Dual use research of concern
☒	☐ Plants

Methods

n/a	Involved in the study
☒	☐ ChIP-seq
☒	☐ Flow cytometry
☒	☐ MRI-based neuroimaging

Plants

Seed stocks

Report on the source of all seed stocks or other plant material used. If applicable, state the seed stock centre and catalogue number. If plant specimens were collected from the field, describe the collection location, date and sampling procedures.

Novel plant genotypes

Describe the methods by which all novel plant genotypes were produced. This includes those generated by transgenic approaches, gene editing, chemical/radiation-based mutagenesis and hybridization. For transgenic lines, describe the transformation method, the number of independent lines analyzed and the generation upon which experiments were performed. For gene-edited lines, describe the editor used, the endogenous sequence targeted for editing, the targeting guide RNA sequence (if applicable) and how the editor was applied.

Authentication

Describe any authentication procedures for each seed stock used or novel genotype generated. Describe any experiments used to assess the effect of a mutation and, where applicable, how potential secondary effects (e.g. second site T-DNA insertions, mosiacism, off-target gene editing) were examined.
